# The Role of Particle Inhalation in Idiopathic Pulmonary Fibrosis

**DOI:** 10.3390/ijms26178736

**Published:** 2025-09-08

**Authors:** Andrew J. Ghio, Rahul G. Sangani, Nevins W. Todd

**Affiliations:** 1 US Environmental Protection Agency, Research Triangle Park, NC 27711, USA; 2 Department of Medicine, West Virginia University, Morgantown, WV 26501, USA; rgs4383@gmail.com; 3 Department of Medicine, University of Maryland School of Medicine, Baltimore, MD 21201, USA; ntodd@som.umaryland.edu

**Keywords:** pulmonary fibrosis, smoking, silica, asbestos, air pollution, iron

## Abstract

Idiopathic pulmonary fibrosis (IPF) is currently defined as a progressive fibrosing interstitial lung disease (ILD) associated with a histopathologic and radiologic pattern of usual interstitial pneumonia (UIP). The relationship between IPF and particles is described, and a pathogenesis for the disease is proposed based on an association with these exposures. In clinical studies and epidemiological investigations, the majority of IPF diagnoses are associated with particle exposures. Cigarette smoking presents the greatest particle challenge in any society, and a relationship with IPF has repeatedly been demonstrated. Environmental exposures to particles other than cigarette smoking, including biomass fuel smoke and ambient air pollution, as well as numerous occupational particle exposures, have also been associated with IPF. The pathogenesis of the disease includes a complexation and sequestration of cell iron at the particle surface, which results in a functional cell deficiency of the requisite metal. In response to the insufficiency of metal in cells, there is the synthesis of biopolymers, including exopolysaccharides (e.g., hyaluronic acid), which accumulate in the extracellular matrix. These biopolymers complex iron and, following depolymerization, facilitate the delivery of the metal intracellularly via receptor-mediated uptake. This process reverses the functional iron deficiency introduced by the particle. Pulmonary fibrosis after particle exposure reflects a response to the modification of a functional intracellular iron deficiency in the lower respiratory tract. The temporal and spatial heterogeneity of IPF results from a dose–response with retained particles and reversibility of the fibrosis.

## 1. Introduction

Asbestos is an industrial term used to denote a group of six fibrous, silicate particles previously of commercial value because of their tensile strength and chemical and heat resistance. These fibrous particles include five amphibole minerals (actinolite, amosite, anthophyllite, crocidolite, and tremolite) and one serpentine mineral (chrysotile). Idiopathic pulmonary fibrosis (IPF) is currently defined as a progressive, fibrosing interstitial lung disease (ILD). This definition suggests an umbrella term that includes pulmonary fibrosis lacking an identified etiology with potentially numerous radiologic and histologic patterns of disease. However, over the past 20 years, the diagnosis of IPF evolved to describe a narrower phenotype of disease, with well-defined clinical, histologic, and radiologic findings [1,2,3,4]. Histologically, the lungs in IPF demonstrate a usual interstitial pneumonia (UIP) pattern of injury, a pattern characterized by several pertinent findings: geographically heterogenous areas of dense fibrosis, interspersed areas of relatively normal-appearing lung architecture, small cysts lined by ciliated respiratory epithelium and often filled with mucin (i.e., honeycombing), and juxtaposed areas of extracellular matrix with spindle-shaped fibroblasts (i.e., fibroblast foci) [1,2,3,5,6]. This UIP pattern of lung injury is generally considered distinct from other histologic patterns of lung injury, such as nonspecific interstitial pneumonia (NSIP), organizing pneumonia (OP), and acute interstitial pneumonia (AIP), which are often observed in patients with alternative clinical forms of ILD.

Radiologically, computed tomography (CT) of the chest in patients with IPF will similarly demonstrate a UIP pattern of lung injury. This can sometimes be unclear since, as noted above, UIP was originally described as a histologic pattern of injury, but over time, the same term has been extrapolated to describe correlative findings on chest CT imaging. The radiologic pattern of UIP is characterized by several pertinent findings: small, linear, irregularly shaped opacities (i.e., reticulations), irregularly shaped and dilated airways (i.e., traction bronchiectasis), and small 3–10 mm cysts in the extreme periphery of the lung (i.e., honeycombed cysts). Relevant for the UIP radiologic pattern, these abnormalities are distributed in peripheral-, posterior-, and basilar-predominant regions of the lung. Similarly to its histologic counterpart, the radiologic UIP pattern is generally considered distinct from other radiologic patterns of lung injury, including non-specific interstitial pneumonitis (NSIP), organizing pneumonitis (OP), and acute interstitial pneumonitis (AIP) observed in patients with alternative forms of ILD.

Clinically, patients with IPF have dyspnea with exertion, but this symptom will occur even at rest as disease progression occurs. Physiologically, pulmonary function testing (PFT) shows a restrictive ventilatory defect due to increased elastic recoil forces in the lung and impaired gas exchange, which is manifested by a low diffusion capacity for carbon monoxide (DLCO). Disease progression varies and is difficult to predict but survival is typically between 2.5 and 5 years [7,8,9,10]. Epidemiological investigation suggests that the incidence of IPF is currently increasing [11].

The histologic, radiologic, and clinical features described above are well recognized by pulmonary physicians and ILD experts, but the current nomenclature for “idiopathic” fibrotic lung diseases can be confusing and misleading [4,9,12]. To date, it has been almost 5 decades since IPF was initially described, and numerous scientific studies (epidemiologic, translational, and molecular investigations) have accumulated demonstrating strong associations between IPF and environmental and occupational inhalational exposures, gastroesophageal reflux disease (GERD), and sporadic and inherited genetic variants. Thus, it is evident that IPF is not an idiopathic disorder, and the term idiopathic pulmonary fibrosis does not seem to accurately represent the currently recognized epidemiologic and genetic features of this disease process [4,12]. Despite these observations, the idiopathic nomenclature, and the term IPF in particular, remains entrenched in pulmonary medicine at the present time. Terms such as IPF phenotype and IPF endotype more accurately describe a spectrum of patients with pulmonary fibrosis that reflects the narrow radiologic and histologic UIP pattern of disease.

The term IPF has been repeatedly employed to describe lung disease after particle inhalation. Those particle exposures associated with IPF in clinical studies and epidemiological investigations are examined. In addition, a pathogenetic basis for a prominent role of particle inhalation in IPF is described.

## 2. Smoking and IPF

**Figure 1:**
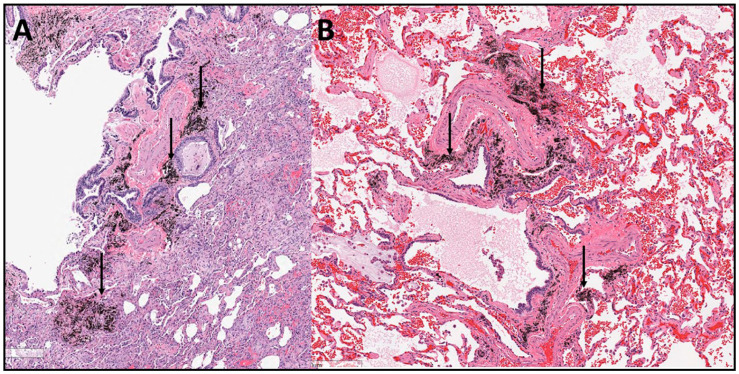
Lung tissue collected from both a smoker diagnosed with IPF (**A**) and a non-smoker exposed to wood smoke particles (**B**) diagnosed with IPF. A pattern of fibrotic lung disease consistent with UIP was observed. Particles (arrows) are evident in both cases. The stain is hematoxylin and eosin, while the magnification approximates 100×.

Cigarette smoking presents the greatest particle challenge to humans, regardless of the state of industrialization in a society. Smoking intermittently exposes an individual to between 10 and 40 mg of particles per cigarette. Pathologic and radiologic findings demonstrated a relationship between smoking and numerous fibrotic injuries and among which are respiratory bronchiolitis, desquamative interstitial pneumonitis (DIP), organizing pneumonitis (OP), nonspecific interstitial pneumonitis (NSIP), and UIP [13,14,15,16,17,18,19,20,21,22,23]. IPF can be a diagnosis of the most advanced fibrotic injury associated with smoking (Figure 1A).

It was recognized decades ago that IPF is more common among ever-smokers relative to lifetime non-smokers. In the initial longitudinal study of patients with UIP, 71% of the cohort were smokers [24]. In other studies of IPF, the prevalence of smoking ranged up to 83% [25]. Cigarette smoking was identified as a potential risk factor in both case–control and observational studies, with the odds ratio ranging from 1.4 to 2.9 for the development of IPF in ever-smokers [26,27,28,29,30,31,32,33,34,35,36,37]. The association was particularly strong among individuals with familial pulmonary fibrosis (FPF), with ever-smokers having an odds ratio of 3.6 for developing disease [38]. Among smokers and ex-smokers, the risk for IPF increased with pack-years of exposure [29,39]. While it was suggested that older studies employed less narrow definitions of IPF, investigations using more recent criteria supported the same associations between (1) smoking and risk for IPF and (2) dose–response between pack-years of smoking and the risk for IPF [37,40]. Smoking influenced the course of disease in IPF, with current smokers developing the disease at a younger age in comparison to non-smokers and ex-smokers [35]. Smoking also increased mortality in IPF patients, with the most common cause of death reported to be the lung disease itself [41,42]. Cigarette smoking was also associated with exacerbations of IPF [43].

Environmental tobacco smoke was reported to be a risk factor for IPF [36]. In addition, individuals exposed to maternal smoking showed an increase in the risk for IPF [40,44].

## 3. Environmental Particle Exposures Other than Smoking and IPF

Environmental particle exposures other than cigarette smoking were also associated with IPF. Fibrotic lung disease resulted from exposures to biomass fuel smoke generated by cooking and heating in inadequately ventilated residences. The presentation included fibrosis called hut lung, domestically acquired particulate lung disease, and bronchial anthracofibrosis associated with ILD. This type of indoor air pollution is a major environmental and public health hazard for large numbers of the underdeveloped world’s population. Analysis of the particles deposited in the lungs by scanning electron microscopy with energy dispersive X-ray spectroscopy determined that it was predominantly carbonaceous with smaller numbers of silica and/or silicate particles [45]. Such exposures to biomass fuel smoke produced fibrotic injury ranging from minimal involvement to UIP, with the latter being consistent with IPF [46,47] (Figure 1B). Imaging in patients exposed to burning of biomass provided evidence of UIP, including honeycombing in subpleural regions, again consistent with IPF [48].

Ambient air pollution, another particle-associated exposure, contributed to the risk for IPF [49]. In Northern Italy between 2005 and 2010, an increment in air pollution was associated with an increase in IPF incidence rate [50]. Long-term exposure to particulate matter with a diameter less than 2.5 microns (PM2.5) elevated the risk of incident IPF [51]. In another study, a relationship between particles in the lung tissue and exposure to air pollution suggested a potential role in IPF [52]. Particle concentration in the air was associated with an increase in the rate of decline of pulmonary function among patients with IPF, also supporting a role for air pollution in disease progression [51,53].

Increased air pollution exposure over the preceding 6 weeks was also associated with an increased risk of acute exacerbation of IPF [54]. Further investigation implicated fine particulate matter as a responsible component [55,56,57]. The “risk-exposure time window” for IPF exacerbation by air pollution particles may lie within 1 to 2 months preceding presentation to medical care providers [58]. Both PM2.5 and NO_2_ were associated with exacerbations of IPF [59].

## 4. Occupational Particle Exposures and IPF

Studies and reviews supported a relationship between occupational particle exposures and IPF [9,10,60,61,62,63,64,65,66]. Initial evidence for an association between occupational exposures and IPF came from an epidemiological investigation, which revealed elevated mortality rates in industrialized areas with exposures to particles [27]. Other studies showed relationships between specific jobs with occupational particle exposures and IPF (Table 1). 

A diagnosis of IPF was two times higher in patients whose jobs exposed them to dust and organic solvents [30]. A case of particle-associated lung injury with a clinical presentation comparable to IPF was observed among workers in a chalk plant [68]. Teachers similarly presented with IPF and demonstrated a significant amount of dust in their lungs [69]. In these cases, silicon dioxide particle was a potential etiologic agent. A fifty percent increase in the risk of developing IPF was seen in patients who were involved in such activities as carpentry work, stone cutting/polishing, bird raising, or employment in a chemical plant, mining, and the insulation industry [67]. A presentation comparable to IPF was associated with agricultural work and farming; this was thought to contribute to over 20% of all cases [31,37,71,73,74,75,76,77]. Farming and gardening, veterinary work, and employment in the metallurgical and steel industry were associated with a pathological pattern of UIP supported by associations of these occupational environments with IPF [70]. Dust-exposed workers showed an early onset of IPF, a longer duration of symptoms at diagnosis, and an association with mortality [78]. By occupational group, the highest IPF mortality rates among females were observed among those farming and fishing, and forestry workers [77].

Studies also correlated specific occupational particles with an increased risk for IPF (Table 2). 

While occupational exposures to dust cause the classical pneumoconioses (e.g., asbestosis, coal worker’s pneumoconiosis, and silicosis), these particles were also associated with IPF [105,106]. A risk of wrongly diagnosing pneumoconiosis as IPF, and conversely, misdiagnosing IPF as pneumoconiosis, was recognized [83]. While infrequent, a histopathologic pattern of involvement reflecting IPF (i.e., a UIP pattern) was observed following asbestos exposure [107,108,109,110,111]. IPF was diagnosed among cases exposed to asbestos identified using electron microscopy techniques [88]. Historic asbestos imports shared a significant, positive linear relationship with IPF mortality [89]. Asbestos exposure increased the incidence of histological UIP [112,113]. Similarly, both coal mine dust and silica exposures were associated with IPF [91,92,93,94]. Deposits of silica/silicates were observed to be increased among IPF patients [83]. Using in-air micro-particle-induced X-ray emission analysis to examine lung tissue, there was a significant difference between IPF and control lungs in terms of silicon, assumed to reflect silica/silicates [114]. Inhaled silicon in the lungs significantly correlated with the annual decline in forced vital capacity in patients with IPF, and a higher accumulation of silicon showed a significantly poorer prognosis. In a study of the total Danish working population (1977–2015), there were increasing incidence rate ratios with increasing cumulative silica exposure for IIPs (which includes UIP reflecting IPF), pulmonary sarcoidosis, and silicosis [115]. In addition, silicates, minerals, mineral dusts, stone and sand dusts, metal dusts (cobalt, hard metals, aluminum, titanium, nickel and magnesium), aluminum trihydrate (Corian) dust, coal, wood fires, organic dusts, textile dusts, vegetable dusts, wood dusts, wood preservatives, animal/livestock dusts, birds, bird and animal feed, mildew, hairdressing, and pesticides have all contributed to IPF diagnoses after occupational exposures [27,29,30,31,33,36,37,61,69,81,84,85,86,87,90,95,96,97,98,99,100,101,102,103,116]. A UIP pattern on CT scan supported IPF in patients who mined uranium, mined tin, and worked with aluminum [117,118,119]. The same UIP pattern on a CT scan also supported IPF after particle exposure to mixed dust [120]. Poorly defined past or current occupational exposures to respirable dusts, dusts, smokes, or chemicals were also associated with IPF [32,36]. Workers exposed to inorganic dust were at the highest risk for IPF mortality [63]. Metal dust and fumes, and organic dust were risk factors for a histopathologic pattern of UIP [70]. Cases of IPF contained greater quantities of silicon and aluminum in lymph nodes compared with controls [81]. Finally, industrial wood smoke exposure was associated with a case presenting with features of IPF [104].

## 5. Synergy of Particle Exposures and IPF

A synergy was observed between particle exposures in increasing the risk for IPF. Such an interaction was noted between smoking and occupational exposures [33]. Heavy smokers at baseline who were exposed to inorganic dusts during their working life had an increased risk of IPF mortality [63]. Exposures to smoking and asbestos interacted to increase IPF risk among those with the minor allele of MUC5B [121].

## 6. Biochemical Pathogenesis of Particle-Associated IPF

**Particle retention in the lung.** The distribution of ventilation following air entry is greater at the bases of the lung relative to the apices due to differences in thoracic ventilation [122,123]. Accordingly, greater numbers of particles are delivered to the lower relative to the upper lung fields. The major pathway for clearance of particles deposited in the conducting airways is the mucociliary escalator, which is efficient and rapid [124]. Ciliated epithelial cells, which participate in this pathway, continue to the terminal bronchiole (TB), leaving the respiratory bronchioles (RBs), alveolar ducts (ADs), and alveoli (i.e., the acinus) removed from the mucociliary escalator. In the acinus, particle clearance includes (1) phagocytosis by alveolar macrophages (AMs), (2) migration of these cells with the particle along the alveolar and bronchiolar surfaces to the TBs, where the mucociliary removal mechanism begins, and (3) transport of the AMs by the moving surface fluid layer [125,126]. AM-mediated clearance is slower than that by the mucociliary escalator in the conducting airways, and particle retention half-times in the distal respiratory tract are subsequently greater [127].

Particle exposure stimulates the production of mediators, which accelerates monocyte release from the bone marrow [128]. Blood monocytes increase in number after particle-associated exposures [129,130,131,132,133]. A release of monocytes from the bone marrow after particle inhalation is followed by their recruitment into the lung with differentiation to macrophages [128,134]. Subsequently, exposure augments the number of macrophages recruited into the lungs, allowing for their increased participation in particle clearance from the distal respiratory tract [134,135]. The effects of gravity on the distribution of blood flow are attributed to the hydrostatic pressure difference between the top and bottom of the lung [122]. Perfusion is greatest in the lower lung fields comparable to ventilation [122]. With a major source of AMs being vascular monocytes, the numbers of these cells present in the lung after particle exposure will accordingly be greater in the lower fields reflecting perfusion [136]. Based on an increased availability of monocyte-derived macrophages to phagocytose particles and expedite their removal, more proficient particle clearance in the lower lung fields is predicted.

The mass flow velocity into the airways approaches zero at the RB, augmenting mechanical deposition of particulate matter at this site [137]. The magnitude of exposure impacts particle clearance from the distal respiratory tract and, as it increases, AM transport of particles from the surfaces of the RBs, ADs, and alveoli to the entry site of the mucociliary escalator (i.e., the TB) is overwhelmed. The RB is that site in the clearance pathway demanding the greatest traverse by these cells originating in the alveoli and destined for the mucociliary escalator at the TB. Following exposures, there results a focal collection of particle-laden AMs in the region of the RB (i.e., respiratory bronchiolitis). With greater particle exposures, there can be a “particle overload” of the distal respiratory tract in which there is a reduction in AM mobility associated with an impairment of clearance [138]. The hallmark of the “particle-overloaded” lung is altered retention kinetics [138,139]. Changes in AM function with “particle overload” have been attributed to augmented particle mass, volume, and/or surface area [139,140]. This is reflected by an excess accumulation of particle-laden AMs frequently observed distributed throughout the RBs, ADs, and alveoli.

An inability of the AMs to eliminate particles from the distal respiratory tract recruits alternative routes or pathways of clearance. The accumulation of AMs in the distal respiratory tract accelerates their migration with phagocytosized particles into the interstitium and subsequent transport to lymphatics, lymph nodes, and pleura [141]. Particle-laden AMs penetrate the pulmonary interstitium [141]. AMs with phagocytosed particles then accumulate in bronchus-associated lymphoid tissue, migrate to peribronchial and perivascular lymphatics, and are transported to regional lymph nodes [141,142,143] and to the pleura [144,145]. Particle numbers increase in the thoracic and retroperitoneal lymph nodes [146]. The lymphatics also transport particles to subpleural sites with access to the pleural space. While there are no direct connections with the lung, specific areas of the parietal pleura absorb and retain high concentrations of particles from the pleural space. The lungs demonstrate particles which, if dark-colored, are appreciated as focal spots of pigment initially in the vicinity of RBs, then in the stroma of the bronchiolar wall and on the surface of adjacent alveoli, and finally over the pleura.

**Particles and the inflammatory/fibrotic response.** The inflammatory/fibrotic response to particles terminates with the end-stage histopathologic pattern of injury recognized as UIP. The pathology demonstrates subpleural and paraseptal fibrosis, fibroblastic foci, and traction bronchiectasis/honeycombing [147]. It is characterized by fibroblastic foci and patchy fibrosis. The radiologist will evaluate the CT scan, which is characterized by the presence of reticulations, traction bronchiectasis, and honeycombing in a basal and peripheral predominant distribution in UIP [1,148].

**Figure 2:**
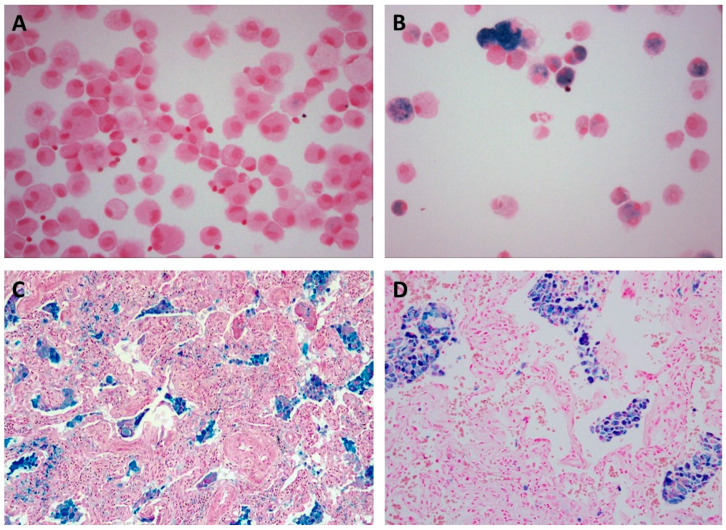
Bronchoalveolar lavage from a healthy non-smoker (**A**) and smoker (**B**) demonstrates a disruption in iron homeostasis of alveolar macrophages with sideromacrophages in the latter. In lung tissue from patients diagnosed with DIP (**C**) and UIP (**D**), macrophages show a disruption in iron homeostasis with sideromacrophages. The stain was Perls’ Prussian blue, while the magnification approximates 100×. (**C**,**D**) are reproduced with permission [157,160].

**Particle-associated IPF and iron.** Particles have surface functional groups which complex metals, including iron [149,150]. Following internalization, the particle will compete for metal utilized in functions critical for cell survival [151]. Particle retention in the lungs is accordingly associated with an accumulation of iron and formation of sideromacrophages (also called siderophages and iron-laden macrophages) supporting a capacity of the particle to complex the metal [152,153,154,155,156,157,158,159,160]. With bronchoalveolar lavage, increased macrophage hemosiderin and iron can be observed among patients with IPF, supporting a disruption of iron homeostasis, and this is frequently associated with particle exposure (Figure 2A,B) [155,156,161,162,163]. In both DIP and UIP, particle-laden macrophages in the distal respiratory tract stain for iron, further supporting the complexation and sequestration of the metal by the particle surface (Figure 2C,D).

Rather than an increased metal availability (or overload), the result of cell exposure to retained particles is a functional iron deficiency because of its sequestration by the particle surface. Evidence confirms a diminished availability of iron following particle exposure [164,165,166]. After loss of its iron to the particle surface, the cell attempts to reverse the deficiency of metal, and this includes an increased expression of proteins involved in its import (e.g., transferrin receptor and divalent metal transporter 1) [167,168,169]. Accordingly, gallium accumulates in the lungs of patients with IPF as the transport of this metal uses transferrin receptor comparable to iron [170]. If either enough iron is complexed by the particle surface or the cell response to increase metal is inadequate, cell survival will be compromised. Cell death associated with iron deficiency following exposure to iron chelators is considered apoptosis [171,172]. Following exposure to compounds/substances which complex/chelate iron, such as particles, cell death was described as apoptosis [173,174]. Findings support active iron sequestration and a cellular response to a functional deficiency in the lungs of smokers and those with disease after particle exposure [151,175,176]. Systemically, metal homeostasis is also altered with particle exposures impacting both anemia of iron deficiency and anemia of chronic disease, reflecting absolute and functional iron deficiencies, respectively [177,178,179,180].

Cell iron deficiency following particle exposures can activate kinases and transcription factors, which are successively associated with a release of inflammatory and fibrotic mediators [150]. Cell exposure to particles activates mitogen-activated protein (MAP) kinases. Activation of the MAP kinase cascade represents a signaling pathway by which exposure to particles mediates biological effects which can be diminished by increasing available iron. Transcription factors control the activity of genes involved in both inflammation, fibrosis, and cell death and are also activated by particle exposure. Comparable to MAP kinases, increased metal availability decreases the activation of transcription factors. Finally, cell exposure to air pollutants produces changes in the expression of mediators (e.g., cytokines and growth factors). Changes in RNA and protein expression for mediators after particle exposure can be diminished by pre-treatment with iron. These results support a relationship between a disruption in iron homeostasis after particle exposure and activation of kinases, phosphatases, and transcription factors and release of mediators which coordinate an inflammatory/fibrotic response.

Pulmonary fibrosis with an accumulation of ECM is a consistent finding in particle-exposed lungs [21,181,182,183]. Components of the ECM bind iron increasing the availability of metal for import to the cell. Several exopolysaccharides in ECM are crosslinked by metals, forming a hydrogel, and participate in iron import [184,185]. Among the exopolysaccharides, polyuronates are a major component of the ECM. Hyaluronic acid is a polyuronate, the most abundant glycosaminoglycan in ECM, which increases with smoking (i.e., a particle exposure), and forms a coordination complex with transition metals, including iron; the coordination is between the metal and carboxyl groups [186,187,188,189]. Subsequently, the hyaluronate receptor CD44 facilitates intracellular iron uptake through this pathway [190,191]. Living systems also utilize polyanionic polysaccharides other than polyuronates to acquire metal [192,193,194]. Individuals with abnormal expression of an exopolysaccharide with an ability to participate in iron uptake may subsequently be at increased risk for pulmonary fibrosis after particle exposure [195,196].

In microbials, complexation by polysaccharides is similarly employed as a pathway for iron import. Polysaccharides in the microbial capsule have abundant uronic acid subunits that participate in metal uptake [197,198,199]. Among metals, uptake is greatest for iron, and large concentrations can be detected in a microbial capsule [198,200]. The availability of iron influences both the production of these polysaccharides and capsule formation [201,202,203,204,205]. Microbials generate biofilms, which include exopolysaccharides (e.g., polyuronates) of varying chain length and composition [206,207]. With biofilm formation, microbes effectively concentrate and utilize metals, with iron being preferred over others [208]. Microbes respond to iron deficiency by using the metal complexed by the components of the biofilm as a “sink”. Removal of iron from a medium increases polyuronate and biofilm production as the microbe attempts to reverse the deficiency [209,210,211]. In contrast, elevated iron concentrations inhibit biofilm formation in a dose-dependent manner [212,213,214,215,216,217]. Accordingly, biofilm production is induced in iron-restricted conditions and is repressed by increased availability of the metal, supporting a role for exopolysaccharides (e.g., polyuronates) in the acquisition of requisite metal comparable to the EPS in IPF associated with particle exposure [214].

**Figure 3:**
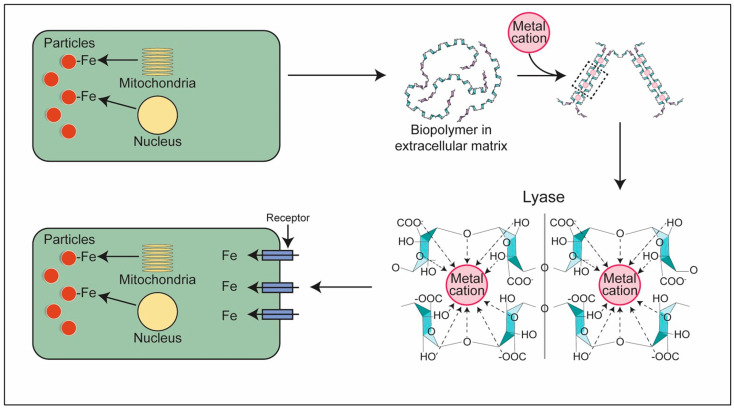
Participation by biopolymers in increasing iron availability to particle-exposed cells. The particle exposure impacts a functional iron deficiency (**top left**). Biopolymer (e.g., polyuronates, collagen, and elastin) is produced in response to a cell functional iron deficiency and complex metal (**top right**). Lyases act to produce oligomers (**bottom right**) which are imported into the cells via receptors thus delivering the metal to reverse the deficiency (**bottom left**).

Comparable to the exopolysaccharides, collagen and its peptides (<10 kDa) bind, complex, and chelate iron as a result of abundant functional groups including carboxylates, hydroxyls, and amines [218,219,220,221]. This interaction of collagen with metals is a recognized method for its stabilization (i.e., tanning which most commonly is achieved with chromium but iron can be employed) [222,223]. It is plausible that both a polymerization and depolymerization of collagen with a cell deficiency of iron provide complexed metal requisite for cell function and survival, comparable to exopolysaccharides. In support of this, the expression of matrix metalloproteinases, a family of extracellular proteases that degrade extracellular cellular matrix, including collagen, can correlate with iron availability [224,225,226,227]. In addition, elastin stains with iron compounds, demonstrating an affinity to complex the metal [21,228,229]. A greater availability of iron to cells decreases the synthesis of these biopolymers (exopolysaccharides, collagen, and elastin) supporting a role for their involvement in metal homeostasis [224,230,231,232]. Metals also participate in a depolymerization of these substances. Following the reaction with iron, the biopolymer is degraded to monomers/oligomers which improves cell metal import [183,233,234]. Accordingly, (1) metal deficiency increases the synthesis of biopolymers included in ECM, (2) these substances complex metal, (3) depolymerization then provides monomers/oligomers to cells via receptor-mediated uptake, and (4) a reversal of the metal deficiency, which initiated the fibrotic response, follows (Figure 3). Pulmonary fibrosis reflects an attempt by the host to modify a functional intracellular iron deficiency after particle exposure.

**Figure 4:**
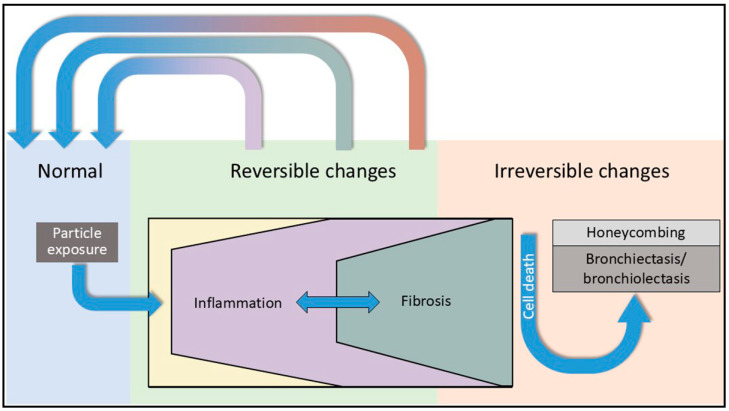
Schematic of inflammation and fibrosis following particle exposure. Both inflammation and fibrosis include reversible changes with a focus on impacting host iron homeostasis and increasing available iron. This can include lung injuries of RB-ILD, DIP, OP, and NSIP. With failure to resolve the disruption in iron homeostasis, the exposure can proceed onto irreversible changes in UIP (e.g., honeycombing).

Fibrosis is reversible, including that in the lung [235,236,237,238,239]. RB-ILD, DIP, OP, and NSIP can reverse reflecting resolution of some portion of the injury [240,241]. As a result of reversibility of ECM deposition, progression of the response may appear heterogeneous with highly involved areas immediately adjacent to normal tissue (i.e., a UIP pattern). Accordingly, spatial and temporal heterogeneity of the fibrotic response to particles can indicate disparities of particle retention in the distal respiratory tract in retention and reversibility of the fibrotic injury (Figure 4).

**Figure 5:**
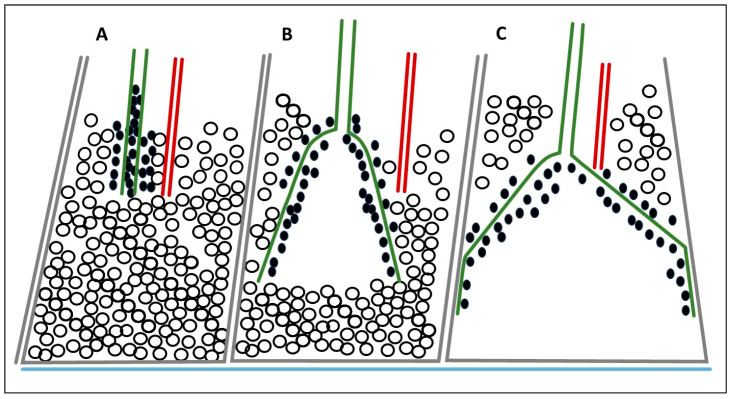
Schematic of bronchiolectasis and honeycombing associated with particle exposure. Cell death in the most distal respiratory units impacts cell death reflected by emphysema and bronchiolectasis. In the periphery of the lower lung fields (blue line is the visceral pleura) the TB/RB (green cylinder) and blood vessel (red cylinder) are located centrally in the secondary lobule with alveoli around it (black circles) (**A**). With a significant particle exposure and retention (black dots), apoptosis will result in widening of the airway, i.e., bronchiolectasis (**B**). With enlargement, the airway meets the wall of the secondary lobule (**C**).

With a failure to resolve an absolute or function cell deficiency of requisite iron, the cell cycle is obstructed and apoptosis initiated. Cell death in the most distal units, i.e., the alveoli, of the lung will result in emphysema [242]. However, apoptosis in tubular structures, such as airways, produces a pattern of tissue injury which includes widening of the cylindrical organization (Figure 5A,C) [243,244,245,246].

After some threshold of particle exposure is exceeded in the airways, bronchiectasis/bronchiolectasis is anticipated. Remodeling of lung tissue after particle exposure can include a continuum of injury which includes “traction” bronchiectasis, bronchiolectasis, and honeycombing. The polyhedral secondary lobule includes 3 to 5 terminal bronchioles separated from other secondary lobules by connective tissue. As the distal bronchiole enlarges with metal insufficiency, it eventually meets the wall of the secondary lobule. Honeycomb cysts (3 to 25 mm) can therefore approximate the diameter of the secondary lobule (10–25 mm) [247]. The wall of the honeycomb cyst will be a summation of the sides of both the airway and the interlobular septa of the secondary lobule. Bronchiectasis/bronchiolectasis, and honeycomb cysts include a spectrum of airway injury with the latter representing the final product (i.e., the UIP pattern observed in IPF following exposure to particle). In support of this, radiographic findings typical of honeycombing and respiratory-lined cysts correspond closely with bronchiolectasis histologically and they appear to be dilated bronchioles and alveolar ducts with apoptotic cells [23,248,249]. Traction bronchiectasis and honeycombing reflect a range of airway remodeling which accompanies inflammation/fibrosis in IIP associated with particle exposure [249]. The cysts in honeycombing areas are covered by cuboidal or even ciliated columnar cells showing an immunohistochemical and molecular bronchiolar phenotype. There is no biomarker.

## 7. Conclusions

Particle exposures can be causative in a majority of IPF cases. In the evaluation of IPF, a history of (1) smoking, (2) environmental tobacco smoke exposure, and (3) other environmental and occupational exposures (e.g., burning of biomass, metals, and inorganic dusts) should be questioned. The number of IPF cases can be reduced through public health policies targeting tobacco cessation and controls of environmental and occupational dust levels [36]. The implementation of policies to control exposures in occupational settings and rural areas, where IPF cases are frequently higher, will be difficult without allocation of additional resources. One of the implications derived from this proposal is that there is a relationship between fibrotic lung injury and iron homeostasis. This would be comparable to other diseases where epidemiology, clinical presentation, treatment, and prognosis can be associated with iron availability (e.g., collagenous esophagitis, collagenous gastritis, cirrhosis, and collagenous colitis in the gastrointestinal tract) [250].

## Figures and Tables

**Table 1:** Occupations associated with increased risk for IPF.

Occupation(s)	Reference(s)
Stone cutting/polishing	[67]
Mining	[67]
Insulating	[67]
Work in a chalk plant	[68]
Teaching	[69]
Metallurgical and steel industry work	[70]
Dentist, dental hygienist, or dental technician	[71,72]
Agricultural work and farming	[31,37,70,71,73,74,75,76,77]
Livestock work	[67,70]
Veterinarians and gardeners	[70]
Carpentry work and woodworking	[67,71]
Hairdresser	[67]
Chemical and petrochemical industries	[67,71]

**Table 2:** Particle exposures are associated with increased risk for IPF.

Exposure(s)	Reference(s)
Respirable dusts, smoke, gases, or chemicals	[32,36]
Inorganic particles	[63,79,80]
Silicon and aluminum	[81]
Silica and silicates	[82,83]
Silica and minerals	[84]
Aluminum silicate	[85]
Chalk/silica and silicates	[68,69]
Stone, sand, or silica	[86]
Stone and sand dust	[87]
Asbestos	[36,82,88,89,90]
Coal	[91]
Silica	[92,93,94]
Mineral dusts	[90]
Metal dusts and fumes	[37,67,70,76,84,87,90,95,96,97,98,99]
Aluminum trihydrate (Corian) dust	[100]
Organic dust	[70]
Vegetable dusts	[67]
Organic dusts	[76]
Organic dust (livestock/agriculture/farming)	[96,101]
Animal dusts	[69]
Animal feeds	[71]
Moulds/birds	[101]
Soil	[31,73,74,75]
Wood dust	[37,73,76,84,97,102]
Wood dusts (birch and hardwood)	[103]
Wood preservatives	[71]
Industrial wood smoke	[104]
Diesel exhaust particles	[30]
Pesticides	[37,71,76]

## Abbreviations

AM	alveolar macrophage
AD	alveolar duct
DIP	desquamative interstitial pneumonitis
ECM	extracellular matrix
IIP	idiopathic interstitial pneumonitis
ILD	interstitial lung disease
IPF	idiopathic pulmonary fibrosis
NSIP	nonspecific interstitial pneumonitis
OP	organizing pneumonitis
PM2.5	particulate matter with a diameter less than 2.5 micron
RB-ILD	respiratory bronchiolitis-interstitial lung disease
RB	respiratory bronchiole
SEM/EDS	scanning electron microscopy with energy dispersive X-ray spectroscopy
TB	terminal bronchiole
UIP	usual interstitial pneumonitis
